# Effectiveness of Pfizer-BioNTech COVID-19 vaccine as evidence for policy action: A rapid systematic review and meta-analysis of non-randomized studies

**DOI:** 10.1371/journal.pone.0278624

**Published:** 2022-12-06

**Authors:** Megan Wallace, Jennifer P. Collins, Heidi Moline, Ian D. Plumb, Monica Godfrey, Rebecca L. Morgan, Doug Campos-Outcalt, Sara E. Oliver, Kathleen Dooling, Julia W. Gargano

**Affiliations:** 1 National Center for Immunization and Respiratory Diseases, Centers for Disease Control and Prevention, Atlanta, Georgia, United States of America; 2 Epidemic Intelligence Service, Centers for Disease Control and Prevention, Atlanta, Georgia, United States of America; 3 Department of Health Research Methods, Evidence and Impact, McMaster University, Hamilton, Ontario, Canada; 4 College of Medicine and Public Health, University of Arizona, Phoenix, Arizona, United States of America; Hawassa University College of Medicine and Health Sciences, ETHIOPIA

## Abstract

In December 2020, an interim recommendation for the use of Pfizer-BioNTech COVID-19 vaccine in persons aged ≥16 years was made under Food and Drug Administration’s Emergency Use Authorization. In preparation for Biologics License Application approval, we conducted a systematic review and meta-analysis to inform the U.S. Centers for Disease Control and Prevention’s Advisory Committee for Immunization Practice’s (ACIP) decision-making for a standard recommendation. We conducted a rapid systematic review and meta-analysis of Pfizer-BioNTech vaccine effectiveness (VE) against symptomatic COVID-19, hospitalization due to COVID-19, death due to COVID-19, and asymptomatic SARS-CoV-2 infection. We identified studies through August 20, 2021 from an ongoing systematic review conducted by the International Vaccine Access Center and the World Health Organization. We evaluated each study for risk of bias using the Newcastle-Ottawa Scale. Pooled estimates were calculated using meta-analysis. The body of evidence for each outcome was assessed using the Grading of Recommendations, Assessment, Development and Evaluation (GRADE) approach. We identified 80 articles, selected 35 for full-text review, and included 26. The pooled VE of Pfizer-BioNTech COVID-19 vaccine was 92.4% (95% CI: 87.5%–95.3%) against symptomatic COVID-19 with moderate evidence certainty (eight studies), 94.3% (95% CI: 87.9%–97.3%) against hospitalization due to COVID-19 with moderate certainty (eight studies), 96.1% (95% CI: 91.5%–98.2%) against death due to COVID-19 with moderate certainty (four studies), and 89.3% (88.4%–90.1%) against asymptomatic SARS-CoV-2 infection with very low certainty (two studies). The Pfizer-BioNTech COVID-19 vaccine demonstrated high effectiveness in all pre-specified outcomes and extended knowledge of the vaccine’s benefits to outcomes and populations not informed by the RCTs. Use of an existing systematic review facilitated a rapid meta-analysis to inform an ACIP policy decision. This approach can be utilized as additional COVID-19 vaccines are considered for standard recommendations by ACIP.

## Background

In the United States, the Food and Drug Administration (FDA) is the regulatory authority for approval of new vaccines. Following regulatory allowance from the FDA (Emergency Use Authorization or licensure), the Centers for Disease Control and Prevention (CDC) makes public health recommendations for vaccine use under advice from the Advisory Committee for Immunization Practices (ACIP), with interim recommendations following Emergency Use Authorization (EUA) and standard recommendations following licensure. In December 2020, the FDA issued an EUA for the Pfizer-BioNTech COVID-19 vaccine and ACIP issued interim recommendations for the use of the vaccine in persons aged ≥16 years [1, 2]. At the time of the EUA, the available body of evidence of benefits and harms consisted of one phase I randomized controlled trial (RCT) [3] and one large endpoint-driven phase II/III RCT [4], which had met the pre-specified number of events and had a median 2 months of follow-up. In the ensuing months, hundreds of millions of doses of the Pfizer-BioNTech COVID-19 vaccine were administered in the United States and worldwide [5, 6].

On August 23, 2021, the FDA approved the biologics license application (BLA) for the Pfizer-BioNTech COVID-19 vaccine for the prevention of COVID-19 in persons aged ≥16 years [7]; subsequently, the ACIP met to consider a standard recommendation for use. The policy question under consideration was, “Should vaccination with Pfizer-BioNTech COVID-19 vaccine (2-doses, intramuscularly, IM) be recommended for persons 16 years of age and older?”. The ACIP COVID-19 Vaccines Work Group framed the policy question by defining the population, intervention, comparison, and outcomes (PICO) of interest. The Work Group defined the population as persons aged ≥16 years, the intervention as two doses of Pfizer-BioNTech COVID-19 vaccine, and the comparison as no COVID-19 vaccine. The outcomes of interest for potential benefits pre-specified by the work group were symptomatic laboratory-confirmed COVID-19, hospitalization due to COVID-19, death due to COVID-19, and asymptomatic SARS-CoV-2 infection. The pre-specified potential harms were serious adverse events and severe or potentially life threatening reactogenicity. To inform ACIP deliberations, a systematic review of the evidence for benefits and harms for Pfizer-BioNTech COVID-19 vaccine was presented to ACIP on August 30, 2021 [8]. The GRADE approach used by ACIP evaluates certainty in estimates of benefits and harms and incorporates data from both RCT and observational studies [9]. The overall methods and results of GRADE were disseminated with the publication of an ACIP policy note [10] shortly after the policy decision. However, detailed methods and results of the meta-analysis performed to support GRADE have not been published.

The assessment of the RCT evidence was previously described in the policy note [10]. Briefly, the available body of evidence from RCTs to inform the PICO question consisted of one Phase I RCT [3] and one large Phase II/III RCT with six months of follow up [11]. These studies provided evidence for most of the outcomes considered, with the exception of asymptomatic SARS-CoV-2 infection (Table 1) [10]. The initial vaccine roll-out under an EUA allowed for a large body of observational evidence to accrue before initial licensure. To incorporate available observational data in the GRADE assessment in the short timeframe required for the policy decision, we performed a rapid systematic review and meta-analysis to characterize the available Pfizer-BioNTech COVID-19 vaccine effectiveness (VE) data for each of the pre-specified beneficial outcomes (Table 1). Although harms were considered in the GRADE assessment, they were not included in the meta-analysis presented here because they were informed by the RCTs and two large post-authorization safety surveillance systems rather than global published and pre-print studies [3, 4, 11]. To provide additional transparency regarding the data synthesis considered by ACIP and to support future application of these methods, here we present the findings of the rapid review and meta-analysis of vaccine effectiveness studies, which was performed for the outcomes of symptomatic COVID-19, hospitalization due to COVID-19, death due to COVID-19, and asymptomatic SARS-CoV-2 infection.

**Table 1:** Summary results from grading of recommendations, assessment, development and evaluation for Pfizer-BioNTech COVID-19 vaccine.

Outcomes	RCT evidence			Observational evidence		
	No. of studies	Measure of effect^1^ (95% CI)	GRADE evidence certainty	No. of studies	Pooled vaccine effectiveness (95% CI)	GRADE evidence certainty
Benefits						
Symptomatic laboratory-confirmed COVID-19	1	91.1 (88.8–93.1)	High	8	92.4 (87.5–95.3)	Moderate
Hospitalization due to COVID-19	1	100 (87.6–100)	Moderate	8	94.3 (87.9–97.3)	Moderate
Death due to COVID-19	1	83.3 (−38.6 to 98.0)	Moderate	4	96.1 (91.5–98.2)	Moderate
Asymptomatic SARS-CoV-2 infection	0	No data	No evidence	2	89.3 (88.4–90.1)	Very low
Harms						
Reactogenicity	2	4.7 (3.8–5.7)	High	0	─	─
Serious adverse events	2	1.0 (0.8–1.2)	Moderate	0^2^	─	─

**Abbreviations:** CI = confidence interval; GRADE = Grading of Recommendations, Assessment, Development and Evaluation; RCT = randomized controlled trial.

^1^The measure of effect was vaccine efficacy for benefits and relative risk for harms.

^2^No data from observational studies were evaluated for serious adverse events overall; however, two safety surveillance systems were evaluated for specific serious adverse events identified during post-authorization surveillance.

## Methods

### Systematic review

In response to the rapidly changing pandemic, we implemented steps to facilitate timely identification and evaluation of the evidence. This systematic review was completed in accordance with the "Preferred Reporting Project for Systematic Reviews and Meta-Analysis (PRISMA) statement," and follows Cochrane rapid reviews methods (S1 Checklist) [12, 13]. Briefly, we identified studies through an ongoing systematic review and screened for eligibility and inclusion in the meta-analysis. We extracted data with a standardized and piloted tool and assessed risk of bias for each study, making final qualitative assessments of the risk of bias. When appropriate, we performed meta-analyses for each outcome and evaluated measures of consistency. We used sensitivity analyses to further explore influence of study characteristics and robustness of pooled VE estimates to inclusion decisions. The certainty of evidence was assessed using GRADE. The systematic review and meta-analysis took place over a three-week period. Article screening began on August 11, 2021 and we continued including available articles through August 20, 2021, with the results presented to ACIP on August 30, 2021.

To expedite the process of identifying relevant literature, we identified observational studies through an ongoing systematic review conducted by the International Vaccine Access Center and the World Health Organization (IVAC/WHO) [14]. Details of search terms and inclusion criteria are available on the IVAC Vaccine Information and Epidemiology Window (VIEW)-hub platform resources page [14]. Given the evolving nature of the COVID-19 pandemic, we included preprints in the available body of evidence for consideration. Thus, articles published in a peer-reviewed journal or posted to a preprint server and captured by the IVAC/WHO systematic review since real-world use of COVID-19 vaccines began in December 2020 through August 20, 2021 were considered for inclusion. In addition, efforts were made to obtain additional relevant data by hand-searching reference lists and consulting with subject matter experts. Following input from the ACIP COVID-19 Vaccines Work Group, we made *a priori* decisions to further restrict the studies retrieved from the IVAC/WHO systematic review and other sources to the population, intervention, comparison, and outcomes being considered by ACIP. We included studies of the general population and sub-populations (e.g., healthcare workers, persons aged ≥65 years). Inclusion of a given study required assessment of the pre-specified beneficial outcomes starting a minimum of 7 days after the 2nd dose of Pfizer-BioNTech. We included case-control, test-negative, and cohort study designs, and did not discriminate on the basis of SARS-CoV-2 variant or dose interval. We excluded estimates of VE that could not be verified as Pfizer-BioNTech specific (e.g., “mRNA vaccine VE”). Two reviewers cross-validated the IVAC summary tables independently and in duplicate for eligibility. Studies with conflicting reviewer opinion were adjudicated through group discussion.

We extracted data for all relevant outcomes (i.e., symptomatic COVID-19, hospitalization due to COVID-19, death due to COVID-19, and asymptomatic SARS-CoV-2 infection) from the eligible studies. One reviewer performed a full data extraction on each study with a standardized, pilot tested tool; each extraction was reviewed and confirmed by a second reviewer. Information extracted included country, dates, population, dosing interval (standard, defined as consistent with the intervals used in an RCT; or extended, defined as a recommendation for a longer interval than was evaluated in an RCT), circulating variants, study design, publication status, raw numerators and denominators when available, adjusted VE and 95% confidence intervals, and variables used for adjustment. VE was extracted as estimated by the authors using a variety of study designs and analytical approaches based on relative effects, generally defined as 100% x (1-relative risk); relative risk measures included risk ratio, rate ratio, odds ratio, and hazard ratio.

All observational studies were evaluated by two reviewers for study limitations (risk of bias) using the Newcastle-Ottawa Scale (NOS) [15], a 9-point instrument that assesses study limitations related to selection, comparability, and assessment of outcome (cohort) and ascertainment of exposure (case-control or test-negative design). We used the NOS evaluations to consider the entire body of evidence for risk of bias as is assessed in the GRADE process. Studies with scores <7 were considered to have serious study limitations that would have resulted in concerns for risk of bias for the body of evidence. We adjudicated conflicting NOS scores which were qualitatively different (i.e., one score <7 and one score ≥7) through group discussion to determine if a study would be classified as having serious study limitations.

### Meta-analysis

We calculated pooled adjusted VE estimates and 95% confidence intervals using random effects (>3 studies) or fixed effects (≤2 studies) meta-analysis (R Version 4.1.1, meta package) [16]. When multiple studies provided estimates based on the same study population (or a subset), we selected the study with the broadest study population or longest duration of follow-up for inclusion in the pooled estimate. For studies that provided multiple or variant-specific estimates, the overall estimate was used when available. If an overall estimate was not available, we used the VE estimates specific to the Delta or Beta variants, as these were considered most likely to impact VE and Delta was the circulating variant at the time of the policy discussion. Studies with serious limitations as assessed by the NOS were excluded *a priori* from the primary pooled VE assessed used in GRADE. We generated forest plots of VE estimates from all included and excluded studies and forest plots of the pooled VE estimates along with VE estimates from studies included in the pooled estimate.

We performed a series of sensitivity analyses to assess the influence of study characteristics (e.g., special populations vs. general population, preprint vs. peer-reviewed manuscript, standard (3-week) vs. extended dosing interval, study design, study limitations, and circulating variants) on the pooled VE estimates, as indicated by the characteristics of the body of evidence for each outcome. Studies excluded from the primary pooled analysis were included in sensitivity analyses exploring heterogeneity due to study limitations, variant-specific estimates, and publication bias. Heterogeneity was additionally explored using I^2^ statistic. I^2^ values of 75% or greater are generally considered to indicate high heterogeneity [17]. We generated forest plots of the pooled VE estimate from the primary meta-analyses along with estimates generated from sensitivity analyses.

### GRADE assessment

We performed a GRADE assessment of the quality of the evidence for each outcome. GRADE methods dictate that the body of evidence from observational studies is initially classified as low certainty, is then rated down or, if no reasons for lowering certainty, may be evaluated for factors which would increase certainty [18]. We evaluated the available body of evidence for factors that may decrease the evidence certainty (i.e., risk of bias, indirectness, inconsistency, imprecision, and publication bias), and other considerations such as large or very large effect, dose response gradient, and opposing residual confounding which may increase the evidence certainty.

## Results

**Fig 1:**
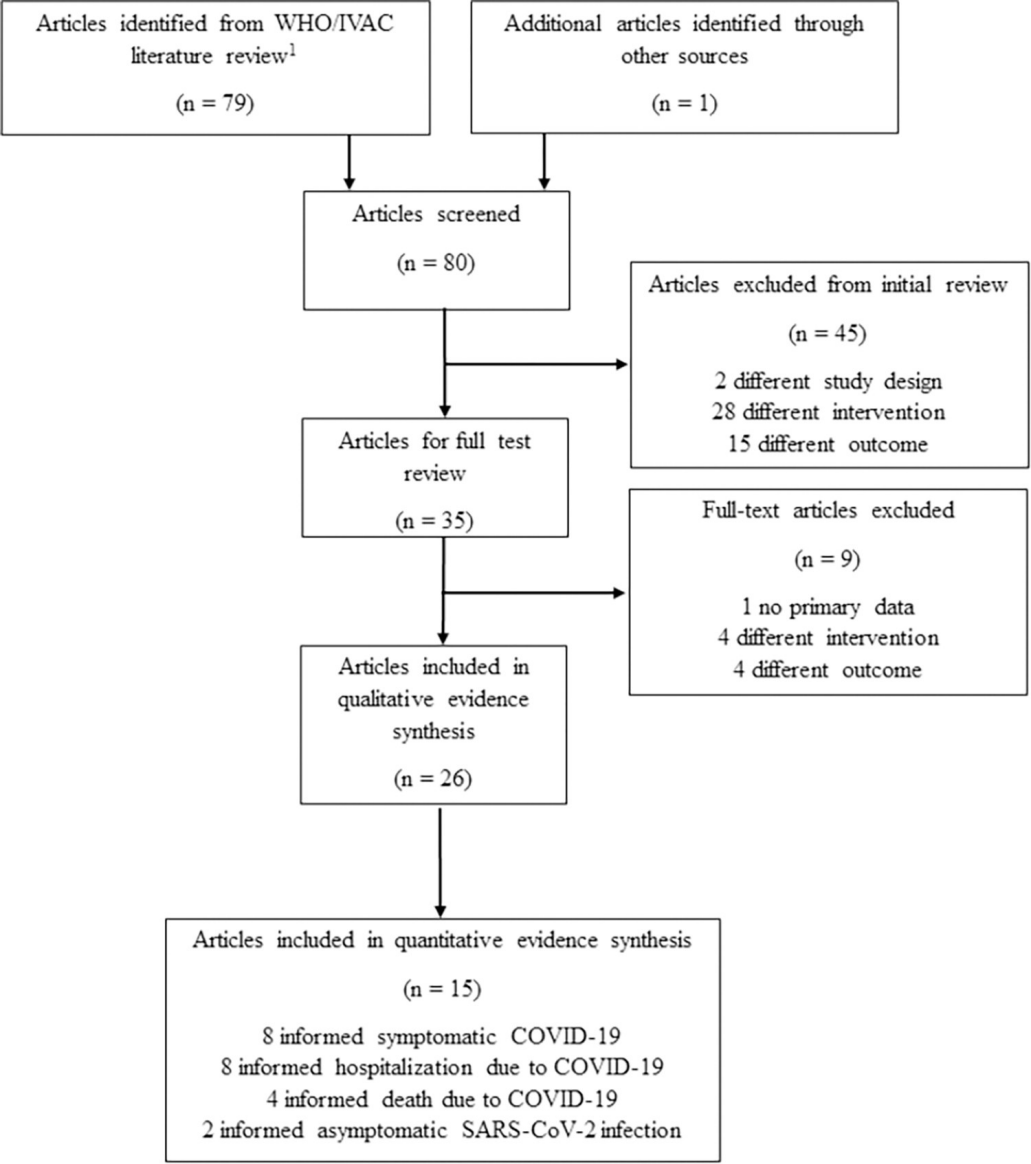
Evidence retrieval and article selection.

After screening 80 studies from the IVAC/WHO review, we identified 35 observational studies that met the PICO criteria for the policy question eligible for full-text review (Fig 1). Of these, we excluded one for having no primary data, four for assessing a different intervention (i.e., a different vaccine or a class of vaccines), and four for assessing a different outcome (e.g., any infection instead of symptomatic or asymptomatic infection). This left 26 observational VE studies eligible for inclusion in the meta-analyses [19–45].

### Symptomatic, laboratory-confirmed COVID-19

Seventeen studies (ten peer-reviewed and seven preprint) reported VE estimates for symptomatic, laboratory-confirmed COVID-19 (Table 2, S1 Fig) [19–25, 27, 30–35, 37, 39, 43, 45].

**Table 2:** Characteristics of studies eligible for inclusion in pooled analysis of vaccine effectiveness of Pfizer-BioNTech COVID-19 vaccine, by beneficial outcome of interest.

Study [ref]	Study design	Location	Population	Time period	Dosing interval	Vaccine effectiveness (95% CI)	Inclusion in pooled analysis
**Symptomatic laboratory-confirmed COVID-19**							
Alali, pre-print [19]	Retrospective Cohort	Kuwait	Healthcare workers	12/24/20–6/15/21	Standard	94.5 (89.4, 97.2)	Included in primary pooled estimate for GRADE
Angel, 2021 [20]	Retrospective Cohort	Israel	Healthcare workers	12/20/20–2/25/21	Standard	97 (94, 99)	Population subgroup of included study
Balicer, pre-print [21]	Prospective Cohort	Israel	Pregnant women	12/20/21–6/3/21	Standard	97 (91, 100)	Population subgroup of included study
Carazo, pre-print [23]	Test-Negative Design	Canada	Healthcare workers	1/17/21–6/5/21	Extended^1^	92.2 (87.8, 95.1)	Included in primary pooled estimate for GRADE
Chung, 2021 [24]	Test-Negative Design	Canada	General population ≥16 years	12/14/20–4/19/21	Extended^1^	91 (88, 93)	Included in primary pooled estimate for GRADE
Dagan, 2021 [22, 25]	Retrospective Cohort	Israel	General population ≥16 years	12/20/20–2/14/21	Standard	94 (87, 98)	Population subgroup of included study
Fabiani, 2021 [27]	Retrospective Cohort	Italy	Healthcare workers	12/27/21–3/24/21	Standard	93.7 (50.8–99.2)	Included in primary pooled estimate for GRADE
Haas, 2021 [30]	Retrospective Cohort	Israel	General population ≥16 years	1/24–4/3/21	Standard	97.0 (96.7, 97.2)	Included in primary pooled estimate for GRADE
Kissling, 2021 [31]	Test-Negative Design	Europe (8 countries)	General population ≥65 years	12/10/20–5/31/21	Standard	87 (74, 93)	Included in primary pooled estimate for GRADE
Lopez Bernal, 2021 [32]	Test-Negative Design	England	General population >80 years	12/8/2020–2/19/2021	Extended^2^	85 (79, 89)	Population subgroup of included study
Lopez Bernal, 2021 [33]	Test-Negative Design	England	General population ≥16 years	10/26/20–5/16/21	Extended^2^	Alpha: 93.7 (91.6, 95.3)	Delta estimate included in variant-specific analysis
						Delta: 88.0 (85.3, 90.1)	
Martinez-Baz, 2021 [34]	Prospective Cohort	Spain	Close contacts ≥18 years	Jan–April 2021	Standard	82 (73, 88)	Included in primary pooled estimate for GRADE
Nasreen, pre-print [35]	Test-Negative Design	Canada	General population ≥16 years	12/14/20–5/2/21	Extended^1^	Non-VOC: 93 (88, 96)	Delta estimate included in variant specific analysis
						Alpha: 89 (86, 91)	
						Beta/gamma: 84 (69, 92)	
						Delta: 87 (64, 95)	
Pouwels, pre-print [35]	Longitudinal Household Survey	United Kingdom	General population ≥16 years	12/1/20–8/1/21	Extended^2^	Alpha-dominant period: 97 (96, 98)	Delta estimate included in variant specific analysis
						Delta-dominant period: 84 (82, 86)	
Regav-Yochay, 2021 [39]	Prospective Cohort	Israel	Healthcare workers	12/19/20–3/14/21	Standard	90 (84, 94)	Population subgroup of included study
Tang, pre-print [43]	Matched Test-Negative Design	Qatar	General population ≥16 years	12/21/20–7/21/21	Standard	Delta: 56.1 (41.4, 67.2)	Delta estimate included in variant specific analysis
Whitaker, pre-print [45]	Prospective Cohort	England	General population ≥16 years	12/7/20–6/13/21	Extended^2^	93.3 (85.8, 96.8)	Included in primary pooled estimate for GRADE
**Hospitalization due to COVID-19**							
Balicer, pre-print [21]	Prospective Cohort	Israel	Pregnant women	12/20/20–6/3/21	Standard	89 (43, 100)	Population subgroup of included study
Dagan, 2021 [22, 25]	Retrospective Cohort	Israel	General population ≥16 years	12/20/20–2/1/21	Standard	87 (55, 100)	Population subgroup of included study
Emborg, pre-print [26]	Retrospective Cohort	Denmark	Groups prioritized for vaccination	12/27/20–4/11/21	Standard	93 (89, 96)	Included in primary pooled estimate for GRADE
Flacco, 2021 [28]	Retrospective Cohort	Italy	General population ≥18 years	1/2/21–5/21/21	Standard	99 (96, 100)	Included in primary pooled estimate for GRADE
Goldberg, pre-print [29]	Prospective Cohort	Israel	General population ≥16 years	12/20/20–3/20/21	Standard	94.2 (93.6, 94.7)	Population subgroup of included study
Haas, 2021 [30]	Retrospective Cohort	Israel	General population ≥16 years	1/24/21–4/3/21	Standard	97.2 (96.8, 97.5)	Included in primary pooled estimate for GRADE
Martinez-Baz, 2021 [34]	Prospective Cohort	Spain	Close contacts ≥18 years	Jan–April 2021	Standard	94 (60, 99)	Included in primary pooled estimate for GRADE
Nasreen, pre-print [35]	Test-Negative Design	Canada	General population ≥16 years	12/14/20–5/2/21	Extended^1^	nonVOC: 96 (82, 99)	Included in primary pooled estimate for GRADE
						Alpha: 95 (92, 97)	
						Beta/Gamma: 95 (81, 99)	
Pawlowski, 2021 [36]	Retrospective Cohort	United States	Adult patients of a large health system	2/15/21–4/20/21	Standard	88.8 (75.5, 95.7)	Population subgroup of included study
Puranik, pre-print [38]	Matched Retrospective Cohort	United States	Adult patients of a large health system	Jan–July 2021	Standard	85 (73, 93)	Included in primary pooled estimate for GRADE
Saciuk, pre-print [41]	Retrospective Cohort	Israel	Adult members of a large HMO	1/18/21–4/25/21	Standard	94.4 (93.2, 95.5)	Population subgroup of included study
Stowe, pre-print [42]	Test-Negative Design	England	General population ≥16 years	4/12/21–6/4/21	Extended^2^	Alpha: 95 (78, 99)	Included in primary pooled estimate for GRADE
						Delta: 96 (86, 99)	
Tenforde, 2021 [44]	Test-Negative Design	United States	Hospitalized adults ≥18 years	3/11/21–5/5/21	Standard	84.3 (74.6, 90.3)	Included in primary pooled estimate for GRADE
**Death due to COVID-19**							
Emborg, pre-print [26]	Retrospective Cohort	Denmark	Groups prioritized for vaccination	12/27/20–4/11/21	Standard	94 (90, 96)	Included in primary pooled estimate for GRADE
Flacco, 2021 [28]	Retrospective Cohort	Italy	General population ≥18 years	1/2/21–5/21/21	Standard	98 (87, 100)	Included in primary pooled estimate for GRADE
Goldberg, pre-print [29]	Prospective Cohort	Israel	General population ≥16 years	12/20/20–3/20/21	Standard	93.7 (92.5, 94.7)	Population subgroup of included study
Haas, 2021 [30]	Retrospective Cohort	Israel	General population ≥16 years	1/24/21–4/3/21	Standard	96.7 (96.0, 97.3)	Included in primary pooled estimate for GRADE
Puranik, pre-print [38]	Matched Retrospective Cohort	United States	Adult patients of a large health system	Jan–July 2021	Standard	100 (-60, 100)	Included in primary pooled estimate for GRADE
Saciuk, pre-print [41]	Retrospective Cohort	Israel	Adult members of a large HMO	1/18/21–4/25/21	Standard	84.0 (76.6–89.1)	Population subgroup of included study
**Asymptomatic SARS-CoV-2 infection**							
Angel, 2021 [20]	Retrospective Cohort	Israel	Healthcare workers	12/20/20–2/25/21	Standard	86 (69, 93)	Population subgroup of included study
Haas, 2021 [30]	Retrospective Cohort	Israel	General population ≥16 years	1/24/21–4/3/21	Standard	91.5 (90.7, 92.2)	Included in primary pooled estimate for GRADE
Pouwels, pre-print [37]	Longitudinal Household Survey	United Kingdom	General population 18–64 years	12/1/20–8/1/21	Extended^2^	Delta-dominant period: 74 (69, 78)	Included in primary pooled estimate for GRADE
Regav-Yochay, 2021 [39]	Observational (Prospective Cohort)	Israel	Healthcare workers	12/19/20–3/14/21	Standard	72 (48, 86)	Population subgroup of included study
Tang, pre-print [43]	Matched Test-Negative Design	Qatar	General population	12/21/20–7/21/21	Standard	Delta: 35.9 (11.1, 53.9)	Included in sensitivity analysis including studies with limitations

^1^Recommended at a maximum interval of 16 weeks between doses

^2^Recommended at a maximum interval of 12 weeks between doses

Of the 17 studies, five were conducted in Israel [20, 21, 25, 30, 39]. The largest Israeli study, by Haas et al., was a nationwide retrospective cohort study evaluating VE from January 24 through April 4, 2021 [30]; this was the only Israeli study included in the primary meta-analysis. An additional retrospective cohort study reporting VE in the general population was excluded from the meta-analysis because it had an overlapping study population and less comprehensive study period (December 20, 2020 through February 14, 2021) [22, 25]. Three cohort studies conducted in specific populations (two in healthcare workers and one in pregnant women) were excluded from the primary meta-analysis because the substantial population and observation period overlapped with the included study [20, 21, 39].

We included two of three studies conducted in Canada, where COVID-19 vaccines were administered at extended dosing intervals (recommended at up to 4 months between doses) [23, 24, 35, 46]. One was a test-negative design study among healthcare workers in Quebec with an observation period from January 17 through June 5, 2021, which was included in the pooled analysis [23]. The other two studies evaluated the same population in Ontario, both using a test-negative design. [24, 35]. The first, Chung et al., had an observation period from December 14, 2020 through April 19, 2021 and provided an overall VE estimate [24]. The second, Nasreen et al, had an observation period form December 14, 2020 through May 2, 2021 and provided variant-specific VE estimates [35]. Given the availability of an overall VE estimate, we used Chung et al. in the primary meta-analysis, but included the variant specific VE estimates from Nasreen et al. in sensitivity analyses.

Of four studies conducted in the United Kingdom or England, we included two in the primary meta-analysis. The first was a community-based survey among a representative sample aged ≥18 years across the entire United Kingdom reporting variant-specific VE estimates, with an observation period from December 1, 2020 through August 1, 2021. We included the Delta-specific estimate from this study in the pooled analysis [37]. Three studies from England, where the recommended dosing interval was 3–12 weeks, reported on data from the National Health Service [32, 33, 45, 47]. We included one of these studies in the primary meta-analysis; a prospective cohort study reporting an overall VE estimate from December 7, 2020 through June 13, 2021 [45]. Two studies with a test-negative design were excluded from the primary analysis: one because it was a subpopulation of an included study [32], and the second because it reported only variant-specific VE estimates [33].

Three other studies with non-overlapping study settings were included in the meta-analysis. A test-negative design study included in the primary meta-analysis followed persons aged ≥65 years from eight European countries from December 10, 2020 through May 31, 2021 [31]. We included one prospective cohort study among close contacts of persons with COVID-19 in Spain, which had an observation period from January through April 2021 [34], and one retrospective cohort study among healthcare workers in Kuwait had an observation period from December 24, 2020 through June 15, 2021 [11]. One retrospective cohort study among the general population in Qatar was identified as having study limitations regarding selection and comparability and was included only in sensitivity analyses [43].

**Fig 2:**
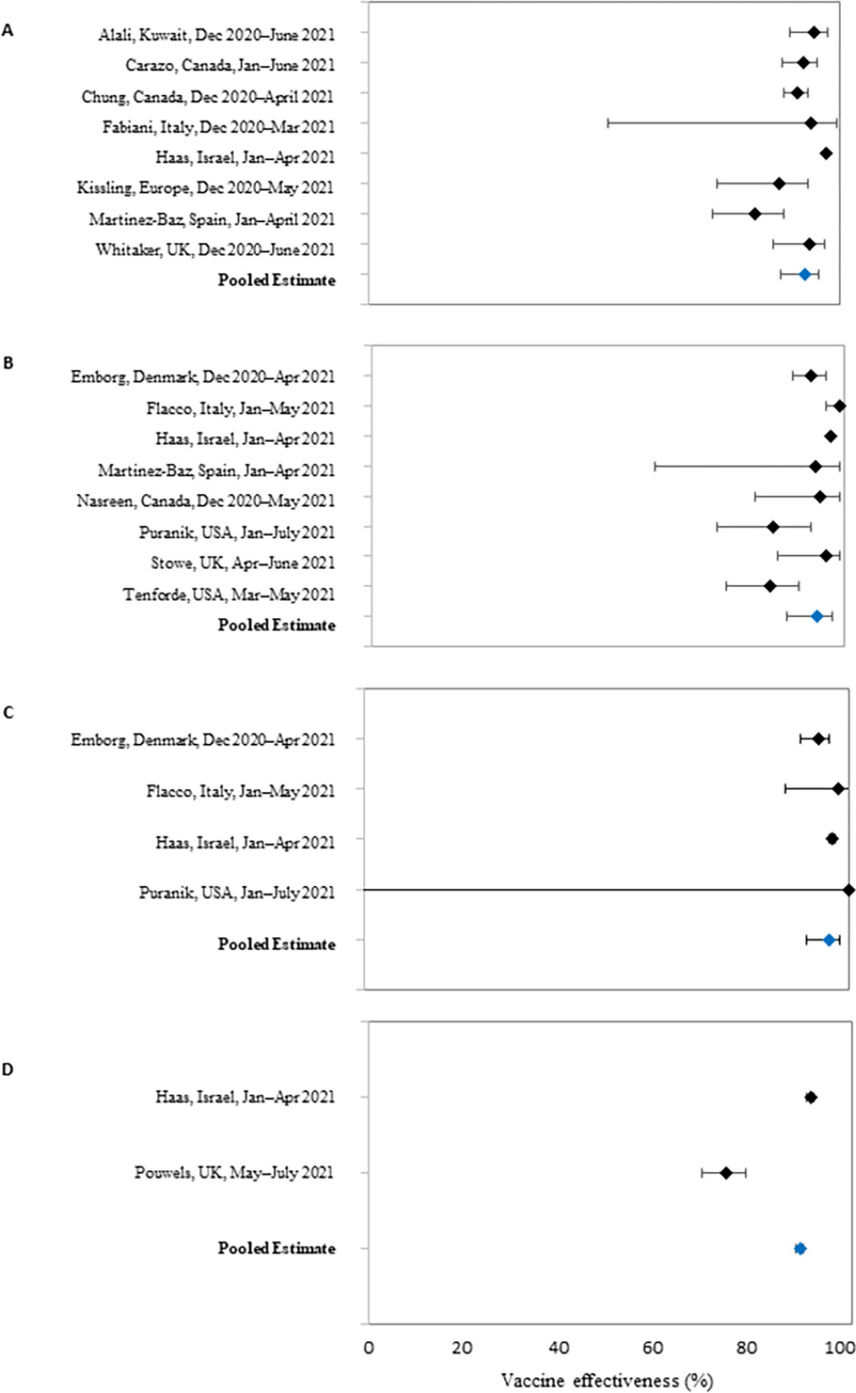
Forest plots showing individual-study and pooled vaccine effectiveness estimates against (A) symptomatic PCR-confirmed COVID-19; (B) hospitalization due to COVID-19; (C) death due to COVID-19; and (D) asymptomatic SARS-CoV-2 infection. Pooled vaccine effectiveness estimates were derived from random effects meta-analyses (A-C) or fixed effects meta-analysis (D). Values for pooled effectiveness estimates and heterogeneity are shown in supplementary tables.

Once studies with duplicate populations (n = 8) or study limitations (n = 1) were excluded, the remaining eight studies included in the primary analysis had a range of VE estimates from 82%–97.0%. The resulting pooled VE estimate for the effectiveness of the Pfizer-BioNTech COVID-19 vaccine in prevention of symptomatic, laboratory-confirmed COVID-19 from a random effects meta-analysis was 92.4% (95% CI: 87.5%–95.3%) (Fig 2A). The VE estimates from all 17 studies available ranged from 56.1%–97% (Table 2, S1 Fig).

**Fig 3:**
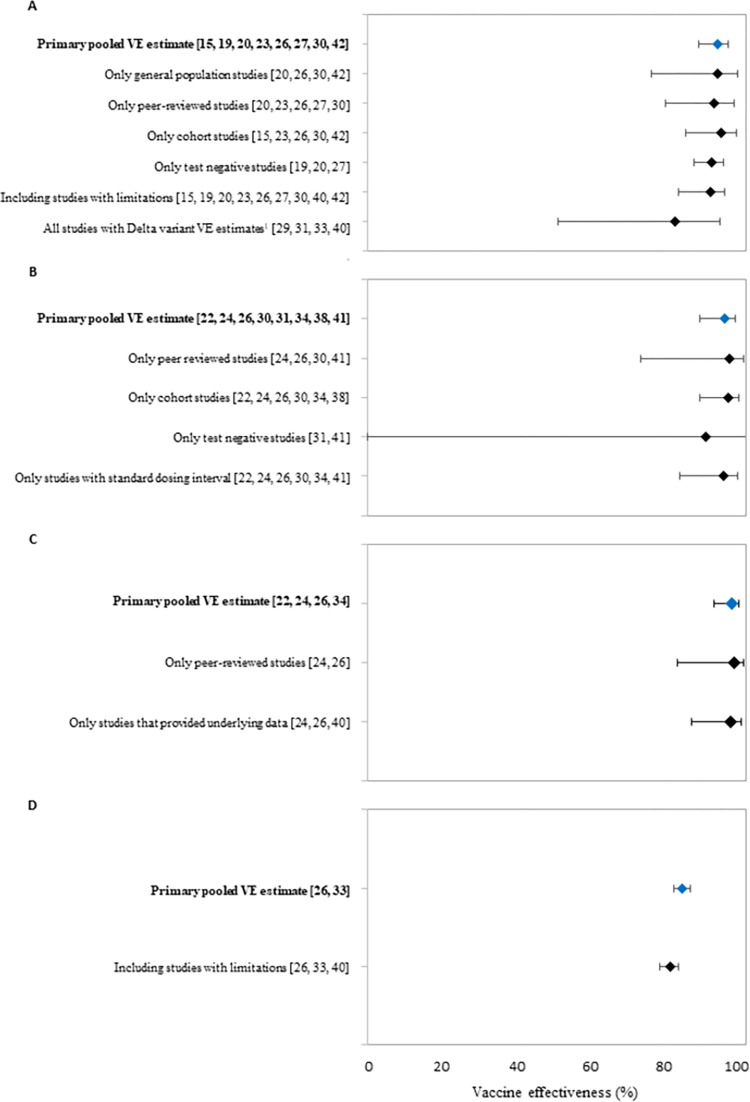
Forest plots showing primary pooled vaccine effectiveness estimates and estimates from sensitivity analyses for (A) symptomatic PCR-confirmed COVID-19; (B) hospitalization due to COVID-19; (C) death due to COVID-19; and (D) asymptomatic SARS-CoV-2 infection. Pooled vaccine effectiveness estimates were derived from random effects meta-analyses (A-C) or fixed effects meta-analysis (D). Values for pooled effectiveness estimates and heterogeneity are shown in supplementary tables. ^1^Three studies provided a Delta-specific estimates [29, 31, 40] and one provided an estimate for the Delta-dominant period [33].

Sensitivity analyses for symptomatic, laboratory-confirmed COVID-19 resulted in pooled VE estimates ranging from 81.2%–93.5% (Fig 3A, S1 Table). Most sensitivity analyses yielded VE estimates similar to the primary pooled VE estimate used for GRADE. However, a meta-analysis of the four studies that provided Delta-specific estimates resulted in a VE that was somewhat lower (VE: 81.2%; 95% CI: 50.2%–92.9%).

### Hospitalization due to COVID-19

We identified 13 observational studies that reported Pfizer-BioNTech VE estimates for hospitalization due to COVID-19 (Table 2, S1 Fig) [21, 25, 26, 28–30, 34–36, 38, 41, 42, 44]. Of these, six were peer-reviewed and seven were pre-print articles.

Five studies were from Israel [21, 25, 29, 30, 41], of which three [21, 25, 30] were previously described. Two additional, general population cohort studies provided VE estimates, one with an observation period from December 20, 2020 through March 20, 2021 and the other from January 18 through April 25, 2021 [29, 41]. As with the outcome of symptomatic COVID-19, we only included Haas et al., from the Israeli studies in the primary meta-analysis for COVID-19 hospitalization as it represented the broadest population and had substantial population overlap with the other studies.

Three studies provided data from the Unites States [36, 38, 44]. Two were retrospective cohort studies from the same large health network; Pawlowski et al., with an observation period from February 15 through April 20, 2021 [36] and Puranik et al., with an observation period from January through July 2021 [38]. Of these, we included Puranik et al., because of the longer duration of follow up and fewer concerns related to selection. Additionally, we included a case-control study among hospitalized adults aged ≥18 years with an observation period from March 11 through May 5, 2021[44].

We included one retrospective cohort study from Denmark, which reported on populations prioritized for vaccination, with an observation period from December 27, 2020 through April 11, 2021 [26]. A retrospective cohort from Italy provided a VE estimate from the general population aged ≥18 years with an observation period from January 2 through May 21, 2021 [28]. We included the VE estimate from the previously described study from Spain [34] and the Beta/Gamma specific VE estimates from a previously described study from Canada [34, 35]. One study from England, with a test-negative design and extended dosing interval provided variant-specific VE estimates from April 12, 2021 through June 4, 2021 [42]. We included the Delta-specific estimate in the meta-analysis.

The eight included studies had Pfizer-BioNTech COVID-19 VE estimates against hospitalization due to COVID-19, which ranged from 84.3%–99% (Table 2, S1 Fig); the pooled primary VE estimate for hospitalization due to COVID-19 was 94.3% (95% CI: 87.9–97.3) (Fig 2B). Five studies (VE estimate range: 87%–94.4%) were not included in the primary meta-analysis because they reported on the same study population as an included study. Sensitivity analyses resulted in pooled VE estimates ranging from 89.4%–95.7% (Fig 3B, S1 Table).

### Death due to COVID-19

Six studies (two peer-reviewed, four preprint) reported estimates of VE by Pfizer-BioNTech vaccine against death due to COVID-19 (Table 2, S1 Fig) [26, 28–30, 38, 41]. We included previously described studies from Denmark, Italy, and the United States in the primary meta-analysis for this outcome [26, 28, 38]. Three previously described studies from Israel provided VE estimates, but only the estimate from Haas et al. was included because of population overlap (VE estimate range of excluded studies: 84.0–93.7). The four studies included in the primary meta-analysis for death due to COVID-19 had VE estimates ranging from 94%–100% (Table 2, S1 Fig). The resulting pooled VE estimate from a random effects meta-analysis was 96.1% (95% CI: 91.5%–98.2%) (Fig 2C). Sensitivity analyses resulted in pooled VE estimates ranging from 95.6%–96.8% (Fig 3C, S3 Table).

### Asymptomatic SARS-CoV-2 infection

Five previously described studies reported Pfizer-BioNTech VE estimates for asymptomatic SARS-CoV-2 infections: three from Israel, one from Qatar, and one from the United Kingdom (Table 2, S1 Fig) [20, 30, 37, 39, 43].

Among the Israeli studies, one study [39] was considered to have serious study limitations due to selection of controls and comparability of asymptomatic cases and controls. The two other Israeli studies all had population overlap with Haas et al., which was included in the pooled analysis. The study from Qatar was not included in the pooled analysis because of study limitations related to selection of controls, and comparability of asymptomatic cases and controls [43]. The community-based survey from the United Kingdom reported VE estimates by variant dominant period [37]. We included the VE estimate for the Delta-dominant period in the meta-analysis. In total, we excluded three studies from the meta-analysis, two because they reported on the same study population as an included study and one due to study limitations.

A fixed effects meta-analysis using the remaining two studies resulted in a pooled VE estimate of 89.3% (95% CI: 88.4%–90.1%) (Fig 2D). In a sensitivity analysis which included the study considered to have study limitations, the pooled VE estimate was 88.1% (95% CI: 87.2%–89.0%) (Fig 3D).

### GRADE

We did not downgrade for risk of bias because we required studies to have an NOS of ≥7 for inclusion in the primary meta-analyses. We did not downgrade for indirectness because our inclusion criteria specified that studies must address the PICO defined policy question for inclusion in the meta-analysis and that the follow-up duration was deemed adequate for all included studies. Regarding inconsistency, I^2^ values (see S1–S4 Tables) and methodologic differences raised concern for possible heterogeneity. However, estimates from the included studies all indicated a substantial benefit from vaccination, with overlapping confidence intervals, for all outcomes except asymptomatic SARS-CoV-2 infection; this was the only outcome downgraded for serious inconsistency. We did not downgrade for imprecision because the pooled analyses for each outcome consisted of hundreds of thousands of persons and thousands of events, resulting in precise estimates. The pooled VE estimates for symptomatic COVID-19, hospitalization due to COVID-19, and death due to COVID-19 had no concerns for rating down and showed large effect associations, and the body of evidence for each of these outcomes was rated up to moderate certainty. The certainty of evidence for asymptomatic SARS-CoV-2 infection was rated very low evidence due to serious concern for inconsistency (Table 1).

## Discussion

In this rapid systematic review and meta-analysis performed to inform policy discussions for ACIP, the Pfizer-BioNTech COVID-19 vaccine demonstrated effectiveness in the prevention of symptomatic, laboratory-confirmed COVID-19 (VE: 92.4%), hospitalization due to COVID-19 (VE: 94.3%), death due to COVID-19 (VE: 96.1%), and asymptomatic SARS-CoV-2 infection (VE: 88.1%) when compared with receipt of no COVID-19 vaccine. These estimates each had a moderate level of evidence certainty based on a GRADE assessment, except for asymptomatic SARS-CoV-2 infection, which had very low evidence certainty. While this review only represents the data available at a single point of time (i.e., the body of evidence available for decision-making at the time of the August 20, 2021 data cutoff prior to the policy vote on August 30, 2021), the estimates were robust to a variety of sensitivity analyses, all of which supported vaccine benefit for each pre-specified outcome.

For the outcomes included in Phase II/III RCT, the pooled VE estimates from the meta-analyses were well aligned with the RCT estimates as shown in Table 1; additionally, the certainty was judged to be the same from RCT and observational evidence for hospitalization and death due to COVID-19 [10]. Furthermore, the observational data included in the GRADE assessment provided additional complementary, sequential and replacement evidence that we did not identify from the RCT data, including observation time in the Delta-dominant period and inclusion of populations excluded from the RCT such as long-term care facility residents and pregnant women [48]. The RCT did not provide data on the outcome of asymptomatic SARS-CoV-2 infection, and the outcomes of hospitalization and death due to COVID-19 had few events during the trial, leading to imprecision based on concerns with fragility in the estimates. The observational studies filled these gaps as they captured many events and provided data for all outcomes [10]. In addition, the observational studies provided ‘real-world’ evidence of the protective effect of vaccines in a variety of populations after routine introduction of the vaccine.

Waning immunity and variants of concern may impact VE over time. The phase II/III RCT showed lower efficacy against symptomatic, laboratory-confirmed COVID-19 ≥4 months post vaccination, however we were unable to calculate VE estimates by the time since vaccination from the available observational data [11]. The phase II/III RCT did not evaluate vaccine efficacy during the Delta-dominant period, and only half of the observational studies included in the primary pooled estimate for symptomatic COVID-19 had any follow up during the Delta-dominant period as identified by the authors. At the time of the systematic review, Omicron had not yet emerged as a variant of concern. Sensitivity analyses of observational studies, which provided Delta-specific VEs or VEs during the Delta-dominant period, showed the Delta-specific estimate for symptomatic COVID-19 was lower than the primary pooled estimate, although Delta variant-specific VE against symptomatic infection was still quite high at 81.2% (95% CI: 50.2–92.9) and had overlapping confidence intervals with the pooled estimate. A single Delta variant-specific VE estimate for hospitalization due to COVID-19 was available, which also showed continued high effectiveness (VE: 96%) [42]. Two studies provided Delta variant-specific VE estimates for asymptomatic SARS-CoV-2 infection with less consistent results (VE range: 36%–74%) [37, 43].

To perform this rapid systematic review and meta-analysis, we built upon existing ongoing efforts in response to the COVID-19 pandemic. The IVAC/WHO systematic review facilitated expeditious identification of relevant articles and the weekly updated summary tables enabled the inclusion of newly identified studies up to the week before the policy discussion. The increased availability of preprint articles aided the inclusion of timely data, which was particularly important in evaluating VE against the Delta variant. We implemented other steps to facilitate a timely systematic review and meta-analysis, including a threshold for the interpretation of the risk of bias, restricting data extraction to only data presented in included studies, and extracting a minimum number of key variables including the adjusted VE measures in each study.

This analysis was subject to several important limitations. The analysis was performed following streamlined methods for the purpose of informing ACIP policy discussions and the analytic decisions made were in that context. No formal protocol was developed and the review was not registered, although *a priori* decisions regarding inclusion and exclusion criteria and the general approach were discussed among an external ACIP Work Group and documented. The specificity of the policy question led to the exclusion of studies that might have provided additional evidence relevant for some of the outcomes. To provide ACIP with the most recent evidence available, we included preprint studies which had not yet been through the peer-review process; however, we assessed each study for limitations using NOS. Use of the NOS tool allowed for rapid assessment of risk of bias, which was further simplified by using NOS ≥7 as a qualitative cut off for serious study limitations leading to exclusion from the primary meta-analysis; however, use of the tool in this way limits the description of the heterogeneity in the risk of bias among the studies. Other, more exhaustive, risk-of-bias tools were not used due to the rapid timeline required for the review [49]. Because of the inclusion of preprints, we did not assess publication bias. To provide ACIP with the most comprehensive body of evidence, we did not exclude studies based on study design, population subgroup, dosing interval, or COVID-19 variant. Despite the limited exclusions, few studies provided estimates of VE against asymptomatic SARS-CoV-2 infection. Pooling estimates from different vaccine effectiveness studies might be limited because of differences between populations and in the way that vaccines were introduced. However, in this context the relative statistical homogeneity, similar effectiveness estimates for most outcomes, and robustness to sensitivity analyses strengthens the pooled estimates.

The publication of this meta-analysis provides transparency in the process and data that informed the ACIP policy decision to recommend Pfizer-BioNTech COVID-19 vaccine. This review evaluated the totality of the data available to provide VE estimates upon which a policy decision could be made. At the time of the ACIP vote, overwhelming evidence of benefits of the Pfizer COVID-19 vaccine was identified, with consistent results from RCTs and observational studies. This is a rapidly evolving field with new studies published weekly, and similar approaches might be beneficial as new observational evidence accrues to inform future COVID-19 vaccine policy decisions. Using the ongoing IVAC/WHO systematic review as the source of studies facilitated a timely review. This rapid meta-analysis process will be repeated as additional COVID-19 vaccines in use under EUAs are licensed and considered for standard recommendations, keeping the *a priori* decisions on inclusion and exclusion criteria and general methods consistent across vaccines and policy questions [50].

## Supporting information

S1 Checklist PRISMA 2020 checklist. (DOCX)

S1 Table Sensitivity analysis for VE of the Pfizer-BioNTech COVID-19 vaccine against symptomatic laboratory-confirmed COVID-19. (DOCX)

S2 Table Sensitivity analysis for VE of the Pfizer-BioNTech COVID-19 vaccine against hospitalization due to COVID-19. (DOCX)

S3 Table Sensitivity analysis for VE of the Pfizer-BioNTech COVID-19 vaccine against death due to COVID-19. (DOCX)

S4 Table Sensitivity analysis for VE of the Pfizer-BioNTech COVID-19 vaccine against asymptomatic SARS-CoV-2 infection. (DOCX)

S1 Fig Forest plots showing primary pooled vaccine effectiveness estimates and estimates from sensitivity analyses for (A) symptomatic PCR-confirmed COVID-19; (B) hospitalization due to COVID-19; (C) death due to COVID-19; and (D) asymptomatic SARS-CoV-2 infection. Pooled vaccine effectiveness estimates were derived from random effects meta-analyses (A-C) or fixed effects meta-analysis (D).
(TIF)

## Abbreviations

ACIP: Advisory Committee on Immunization Practices
BLA: biologics license application
FDA: Food and Drug Administration
GRADE: Grading of Recommendations, Assessment, Development and Evaluation
IVAC: International Vaccine Access Center
WHO: World Health Organization
