# Supplementary material for: Effectiveness of Pfizer-BioNTech COVID-19 vaccine as evidence for policy action: A rapid systematic review and meta-analysis of non-randomized studies
Source: PLoS One. 2022 Dec 6;17(12):e0278624. doi: 10.1371/journal.pone.0278624 (PMC9725157; doi:10.1371/journal.pone.0278624)
Supplement: S1 Table — (DOCX) [file pone.0278624.s002.docx]

**S2 Table. Sensitivity analysis for VE of the Pfizer-BioNTech COVID-19 vaccine against symptomatic laboratory-confirmed COVID-19**

| **Analysis description** | **Pooled VE Estimate (95% CI)** | **I^2^** |
| --- | --- | --- |
| Primary pooled analysis (k=8) | 92.4 (87.5, 95.3) | 95.0% |
| Only general population studies (k=4) | 92.5 (75.0, 97.8) | 97.4% |
| Only peer reviewed (k=5) | 91.6 (78.8, 96.7) | 96.8% |
| Only preprint (k=3) | 93.1 (89.2, 95.6) | 0% |
| Cohort only (k=5) | 93.4 (84.0, 97.3) | 94.8% |
| Test negative only (k=3) | 91.0 (86.1, 94.1) | 0% |
| Standard dosing interval (k=5) | 92.4 (80.2, 97.1) | 95.5% |
| Extended dosing interval (k=3) | 91.6 (88.7, 93.7) | 0% |
| Alpha variant (k=6) | 93.5 (85.8, 97.0) | 95.4% |
| All studies with delta variant estimates (k=4) | 81.2 (50.2, 92.9) | 94.4% |
| Including studies with limitations (k=9) | 90.4 (82.1, 94.4) | 98.0% |
