# Supplementary material for: Effectiveness of Pfizer-BioNTech COVID-19 vaccine as evidence for policy action: A rapid systematic review and meta-analysis of non-randomized studies
Source: PLoS One. 2022 Dec 6;17(12):e0278624. doi: 10.1371/journal.pone.0278624 (PMC9725157; doi:10.1371/journal.pone.0278624)
Supplement: S2 Table — (DOCX) [file pone.0278624.s003.docx]

**S3 Table. Sensitivity analysis for VE of the Pfizer-BioNTech COVID-19 vaccine against hospitalization due to COVID-19**

| **Analysis description** | **Pooled VE Estimate (95% CI)** | **I^2^** |
| --- | --- | --- |
| Primary pooled analysis (k=8) | 94.3 (87.9, 97.3) | 91.7% |
| Only peer reviewed (k=4) | 95.7 (72.3, 99.3) | 93.9% |
| Only pre-print (k=4) | 92.1 (80.6, 96.8) | 52.3% |
| Cohort only (k=6) | 95.2 (87.8, 98.2) | 88.9% |
| Test negative only (k=2) | 89.4^1^ | 60.0% |
| Standard dosing interval (k=6) | 94.0 (82.5, 97.9) | 94.1% |
| Extended dosing interval (k=2) | 95.6 (81.7, 98.9) | 0% |
| Alpha variant (k=7) | 95.3 (89.2, 97.7) | 90.5% |
| Only studies with underlying data provided (k=6) | 92.6 (84.2, 96.6) | 93.8% |

^1^Confidence interval could not be calculated
