# Supplementary material for: Effectiveness of Pfizer-BioNTech COVID-19 vaccine as evidence for policy action: A rapid systematic review and meta-analysis of non-randomized studies
Source: PLoS One. 2022 Dec 6;17(12):e0278624. doi: 10.1371/journal.pone.0278624 (PMC9725157; doi:10.1371/journal.pone.0278624)
Supplement: S3 Table — (DOCX) [file pone.0278624.s004.docx]

**S4 Table. Sensitivity analysis** **for VE of the Pfizer-BioNTech COVID-19 vaccine against death due to COVID-19**

| **Analysis description** | **Pooled VE Estimate (95% CI)** | **I^2^** |
| --- | --- | --- |
| Primary pooled analysis (k=4) | 96.1 (91.5,98.2) | 48.8% |
| Only peer reviewed (k=2) | 96.8 (81.8, 99.4) | 0% |
| Only pre-print (k=2) | 95.6^1^ | 15.1% |
| Only studies with underlying data provided (k=3) | 95.9 (85.6, 98.8) | 63.7% |

^1^Confidence interval could not be calculated
