# Supplementary material for: Effectiveness of Pfizer-BioNTech COVID-19 vaccine as evidence for policy action: A rapid systematic review and meta-analysis of non-randomized studies
Source: PLoS One. 2022 Dec 6;17(12):e0278624. doi: 10.1371/journal.pone.0278624 (PMC9725157; doi:10.1371/journal.pone.0278624)
Supplement: S4 Table — (DOCX) [file pone.0278624.s005.docx]

**S5 Table. Sensitivity analysis for VE of the Pfizer-BioNTech COVID-19 vaccine against asymptomatic SARS-CoV-2 infection**

| **Analysis description** | **Pooled VE Estimate (95% CI)** | **I^2^** |
| --- | --- | --- |
| Primary pooled analysis (k=2) | 83.2 (80.9, 85.3) | 98.1% |
| Including studies with limitations (k=3) | 79.9 (77.3, 82.2) | 98.2 |
