# Supplementary figures and images for: Effectiveness of Pfizer-BioNTech COVID-19 vaccine as evidence for policy action: A rapid systematic review and meta-analysis of non-randomized studies
Source: PLoS One. 2022 Dec 6;17(12):e0278624. doi: 10.1371/journal.pone.0278624 (PMC9725157; doi:10.1371/journal.pone.0278624)

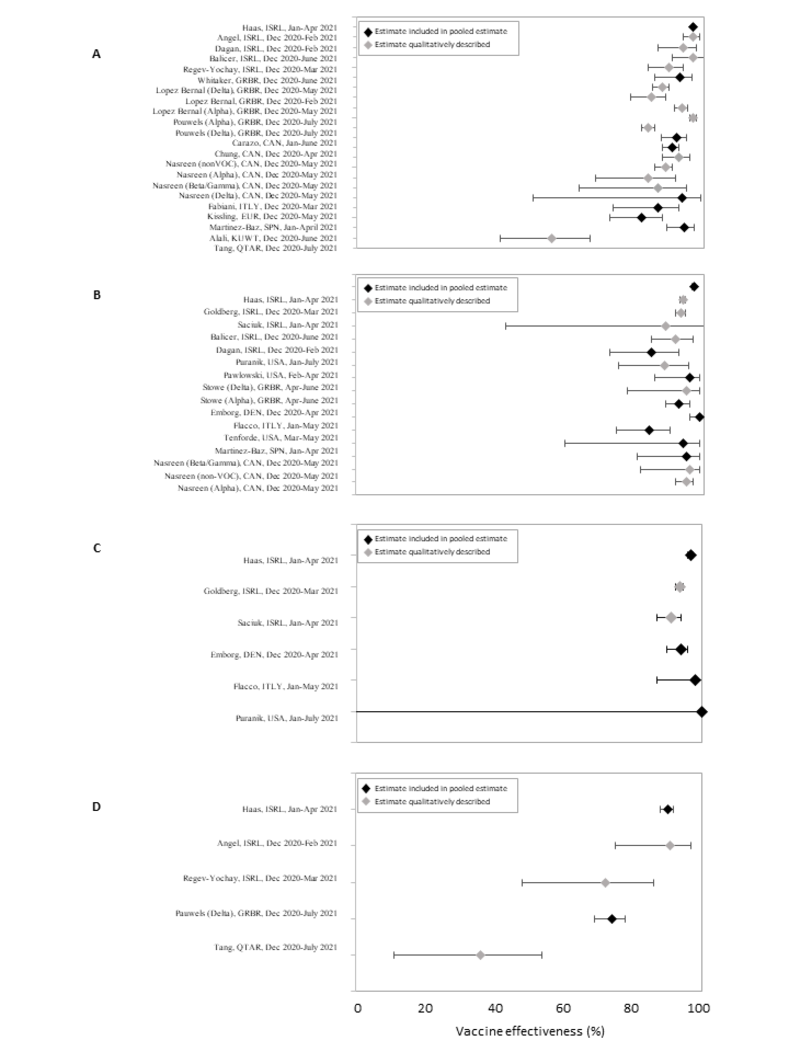

Supplement: S1 Fig — Forest plots showing primary pooled vaccine effectiveness estimates and estimates from sensitivity analyses for (A) symptomatic PCR-confirmed COVID-19; (B) hospitalization due to COVID-19; (C) death due to COVID-19; and (D) asymptomatic SARS-CoV-2 infection. Pooled vaccine effectiveness estimates were derived from random effects meta-analyses (A-C) or fixed effects meta-analysis (D). (TIF) [file pone.0278624.s006.tif]
